# Platform power, athlete branding, generative AI, and the future of sport governance—a systematic review

**DOI:** 10.3389/fspor.2025.1642180

**Published:** 2025-09-15

**Authors:** Hans Westerbeek, Thomas van Schaik

**Affiliations:** ^1^ Institute for Sustainable Industries and Liveable Cities (ISILC), Victoria University, Melbourne, VIC, Australia; ^2^ Victoria University Business School, Melbourne, VIC, Australia; ^3^ The Athlete Brand, Eindhoven, Netherlands

**Keywords:** athlete branding, self-production, disintermediation, digital platforms, generative AI, emerging business models

## Abstract

This systematic review examines how elite athletes are leveraging digital platforms, generative artificial intelligence (AI), and blockchain to build autonomous brands, bypass traditional sport gatekeepers, and develop athlete-owned business models. Drawing on 47 peer-reviewed studies (2016–2025), we synthesise evidence across five domains: athlete branding and self-production, disintermediation, platform-enabled empowerment, AI-driven content innovation, and emerging commercial structures. The findings reveal a decisive shift in sport's power balance, with athletes acting as media producers, cultural influencers, and entrepreneurial actors. Digital platforms enable direct-to-fan engagement, while AI tools lower content production costs whilst personalising interactions and extend global reach. Blockchain facilitates decentralised monetisation and data sovereignty, supporting ventures such as athlete-owned leagues and non-fungible tokens. However, these developments embed new dependencies on platform algorithms and volatile digital markets. From a platform capitalism perspective, athlete autonomy is constrained by corporate-controlled infrastructures; from a value co-creation lens, fan relationships become participatory spaces for shared cultural and commercial value creation. The review highlights governance challenges, including ethical implications of synthetic media, data ownership, and the regulation of AI-enabled branding ecosystems. We argue that sport governance must evolve from a control-oriented model to one that positions athletes as co-creators of value and strategic partners in decision-making. Future research should address equity in digital visibility and sustainable athlete-led business ecosystems. Governance mechanisms that reconcile technological opportunity with autonomy protection should be explored as well. Athletes are no longer peripheral actors in sport's commercial order, they are emerging as its architects, with significant implications for the future of sport governance.

## Introduction

In the digital age, the power dynamics of global sport are undergoing a profound transformation. Once confined to roles defined by clubs, leagues, and federations, elite athletes are increasingly asserting themselves as autonomous media entities, brand strategists, and entrepreneurs. This shift is occurring at the intersection of technological innovation, digital platform economies, and the erosion of institutional control. As a result, the modern athlete is no longer just a performer within the sport industry—they are becoming a central force reshaping it. Over the past decade, academic literature has started to examine athlete branding and digital fan engagement. Yet much of this work has focused on branding as an adjunct to institutional marketing strategies or sponsorship activation within organisationally controlled environments. What remains under-theorised is the disintermediation of the sport business ecosystem: the growing capacity of high-profile athletes to bypass traditional gatekeepers through self-directed branding and content production. The rise of athlete-founded media ventures, direct-to-fan monetisation models, and the early adoption of generative artificial intelligence (AI) tools signal a significant structural shift—one with implications for governance, sponsorship, athlete labour, and the future of sport commercialisation. Recent developments in generative AI, including tools that allow for automated content creation, synthetic media production, and algorithmic fan personalisation, have further amplified athlete autonomy. These technologies are lowering the cost of content production, blurring the line between authenticity and automation, and enabling personalised fan engagement at scale. However, they also raise critical questions about the ethics, regulation, and long-term sustainability of AI-powered brand management in sport. Despite increasing media coverage and scattered empirical examples, no comprehensive academic synthesis has yet explored the interconnected rise of athlete self-production, digital platform empowerment, and AI-enabled business innovation. This gap in the literature limits our understanding of how athlete power is evolving, and what this evolution means for sport management theory, practice, and education. The purpose of this article is to address this gap by systematically reviewing the scholarly literature on athlete branding, athlete-led digital innovation, and the shifting business models of sport. Specifically, the review aims to:

• identify how elite athletes are increasingly acting as independent brand strategists and content producers;
• examine the extent to which athletes are disintermediating traditional sport organisations and building direct commercial relationships with audiences;
• explore the role of digital platforms in amplifying athlete power and influence;
• investigate the use of generative AI in athlete-led branding and communication practices; and
• synthesise evidence of emerging athlete-led business models that challenge established sport industry structures.

By critically analysing peer-reviewed literature from 2016 to 2025, this review develops a conceptual framework for understanding how elite athletes are reshaping the sport business landscape. In doing so, it contributes to advancing theoretical knowledge in sport management and marketing, while also offering practical insights for practitioners navigating this new era of athlete-driven innovation. To interpret and frame the diverse body of literature identified in this review, we propose two perspectives that are relevant—platform capitalism as proposed by Srnicek (1) and value co-creation theory (2) in the next section.

### Conceptual framework

Platform capitalism provides a macro-level understanding of the structural conditions shaping athlete autonomy in the digital sport economy. In this perspective, digital platforms act as intermediaries that extract and monetise data, algorithmically mediate visibility, and concentrate market power in the platform owner's hands. For elite athletes, this framework highlights the tensions between newfound opportunities for direct-to-fan engagement and the risks of dependency on corporate-controlled infrastructures. Value co-creation offers a complementary micro-level lens, focusing on how athletes and stakeholders, including fans, sponsors, and sport organisations, collaboratively generate brand and cultural value. This perspective has been widely recognised in sport management research as a lens for understanding the joint production of value between multiple stakeholders (3, 4). In sport contexts, athletes act not merely as service providers but as co-creators of cultural and commercial value, often engaging directly with fans via digital platforms (5, 6). The service-dominant logic underpinning value co-creation has been adapted in sport to account for the unique interplay of on-field performance, brand symbolism, and fan community dynamics (3). Recent scholarship has also emphasised the role of technology in transforming value co-creation processes, enabling new forms of participatory branding and engagement that are particularly relevant in the era of generative AI (4). Drawing on these frameworks, we conceptualise athlete empowerment as occurring at the intersection of technological opportunities (e.g., AI-enabled content creation, live streaming), actor agency (athletes’ capacity to initiate, control, and monetise brand narratives), and institutional responses (policy, regulation, and governance frameworks). This dual-perspective approach enables us to synthesise findings across the five thematic domains: athlete branding and self-production, disintermediation, digital platforms, generative AI, and emerging business models.

## Method

This study employed a systematic literature review to examine the rising power of elite athletes in the digital age, focusing on athlete-led brand development, digital self-production, and the emergent role of generative artificial intelligence (AI) in sport business innovation. The review broadly followed the PRISMA (Preferred Reporting Items for Systematic Reviews and Meta-Analyses) guidelines. PRISMA guidelines standardise the reporting process for systematic reviews and enhance transparency and reproducibility. In line with these principles, we documented each stage of identification, screening, eligibility assessment, and inclusion of studies (7). The primary aim of this review was to identify, analyse, and synthesise scholarly literature that explores the mechanisms by which athletes are disintermediating traditional sport organisations and increasingly asserting control over their personal brand narratives and monetisation strategies. Key focus areas included the digital transformation of sport branding, athlete-fan engagement via platform economies, and the use of generative AI in athlete-led content and business model innovation. Searches were executed during February and March 2025 by two researchers. Articles were included if they were published in peer-reviewed academic journals; were written in English; were published between 2010 and 2025, with a focus on literature from 2016 onwards; and addressed one or more of the following core themes: athlete branding and self-production; disintermediation in sport business; digital platforms and athlete empowerment; generative AI in branding and content creation and; emerging athlete-led business models. Quality appraisal was undertaken for all included studies using criteria from the Critical Appraisal Skills Programme (CASP) systematic review checklist (8). Each study was assessed for methodological rigour, relevance to the review's aims, and clarity of reporting.

Non-peer-reviewed sources, editorials, and conference abstracts were excluded unless they were cited in peer-reviewed literature and contributed essential conceptual insight. Searches were conducted across five academic databases: Scopus, Web of Science, SPORTdiscus, Business Source Complete, and Taylor & Francis Online. Supplementary searches using Google Scholar were performed to capture grey literature, with peer-reviewed status verified manually. Backward and forward citation tracking was conducted routinely. Search terms combined Boolean operators (to combine and exclude keywords) and topic-specific keywords, such as:

• (“athlete brand” OR “personal branding” OR “athlete entrepreneurship”)
• AND (“self-production” OR “media company” OR “content creation”)
• AND (“social media” OR “digital platform” OR “direct-to-fan”)
• AND (“AI” OR “generative AI” OR “algorithmic content”)
• AND (“disintermediation” OR “sport business innovation”)

**Figure 1:**
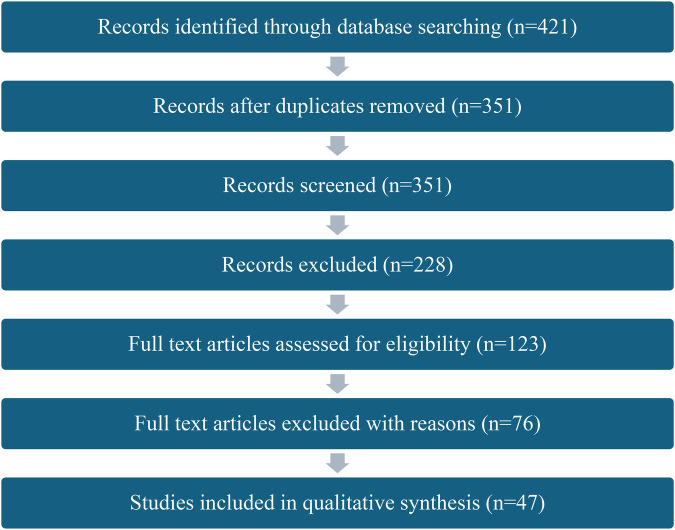
PRISMA flow diagram.

The screening process was conducted in three stages: title and abstract screening, full-text review, and final inclusion. The two reviewers independently screened all records at each stage. Discrepancies were resolved through discussion. Initial searches yielded 421 records. After removal of duplicates 351 records remained. Following title and abstract screening, 228 were excluded. The full text of the remaining 123 articles was assessed for eligibility, resulting in a final selection of 47 studies. This process is illustrated in the PRISMA flow diagram (Figure 1). For each article prior to detailed analysis, a full citation (Harvard style), abstract, and summary of conclusions were recorded. Articles were thematically coded based on relevance to the five focus areas. These themes informed the conceptual synthesis presented in the next sections of the paper starting with a closer look at the emergence of positioning athletes as brands. It needs to be noted that a range of articles that were referenced in regard to the conceptual framework for this paper, were not generated as part of systematic review. This is why the number of references in this paper exceeds the number of studies included in the qualitative synthesis.

## Results

### Athlete branding and self-production

The literature shows that athlete branding in the digital era has shifted from being largely controlled by sports organisations, media handlers, or corporate sponsors to being increasingly athlete-led. Elite athletes have emerged as self-determined brand architects who build, broadcast, and monetise their own narratives. This evolution is documented across multiple studies and is enabled by the convergence of digital accessibility, algorithmic reach, and a growing recognition among athletes of their symbolic and cultural capital. Where once media training and image control dominated the branding playbook, today's athletes operate as content creators and digital entrepreneurs and speaking with voices that can curate culture. As Doyle et al. (9) observe, athlete branding now extends beyond the realm of reputation management to encompass proactive, sustained brand-building efforts across multiple touchpoints. Athletes are no longer just featured in content; they also produce it themselves, determining tone, structure, and storytelling rhythm. They strategically deploy social media, personal websites, streaming platforms, and increasingly, AI-driven content tools. Central to this self-production is the construction of a coherent, authentic narrative. Doyle, Su, and Kunkel (10) found that perceived authenticity, expressed through consistent storytelling, behind-the-scenes access, and unfiltered personal moments, was a key driver of fan engagement on Instagram. Similarly, Kunkel, Doyle, and Na (11) emphasise the strategic use of philanthropy as a form of value signalling, helping athletes align their personal narratives with broader cultural or ethical concerns. Lifestyle branding, especially on platforms like Instagram, plays a pivotal role in this redefinition. Woods et al. (12) conceptualise athlete branding as a three-part construct: centring the self, doing the sport, and being the brand. This model captures the integration of athletic performance, personal identity, and digital storytelling. The role of digital platforms has become foundational. Su et al. (13) demonstrated how specific platform affordances such as interactivity, visibility algorithms, and feedback mechanisms, shape the growth of athlete followings. Their study shows that event-based spikes in attention (during tournaments or championships) offer valuable opportunities to “superboost” social media brands, particularly when athletes can create reactive or topical content in real time (14). In parallel, Na, Kunkel, and Doyle (15) focus on the importance of source credibility and signalling theory in the construction of athlete brand image. Strategic self-presentation featuring credibility cues such as expertise and trustworthiness emerges as a key lever in shaping audience perception. Their research underscores the importance of consistency in messaging and visual identity across platforms. Shreffler, Hancock, and Schmidt (16) reveal how female athletes’ avatar selections reflect conscious branding choices intended to balance femininity with athletic performance. These choices are both strategic and constrained by gendered audience expectations. The gendered dimension of self-production is also evident in Rogstad et al.'s (17) examination of Norwegian female footballers’ Instagram usage during UEFA Women's EURO 2022. Their findings highlight how digital self-presentation is both a site of empowerment and a terrain of negotiation, where athletes curate visibility while managing risk, scrutiny, and algorithmic exposure. The creation of a successful athlete brand relies heavily on emotional labour and the cultivation of intimacy with fans. Kunkel et al. (18) introduce the concept of “self-brand connection” to capture the psychological bond between athlete and fan. Their study demonstrates that athletes who effectively manage their digital presence and align their brand with personal values are more likely to inspire fan loyalty, affective commitment, and advocacy behaviours. Jara Pazmino and Pack (19) investigate the role of social media in facilitating transnational fan relationships among international student-athletes during the pandemic. Their work illustrates how the performative dimensions of branding, especially in moments of crisis or personal vulnerability, can create deep, parasocial engagement across cultural boundaries.

### Disintermediation in sport business

The literature documents a shift in the sport industry toward the disintermediation of traditional structures that historically controlled the whole chain of athletic value creation including athlete representation and commercial activation. Direct-to-fan and direct-to-brand dynamics enabled by digital media have reduced the central role of sport governing bodies and talent agents. Direct employers such as clubs are also trying to come to terms with this loss of control over athletic talent. Athletes, equipped with platform fluency and entrepreneurial intent, now act as media producers and cultural agents, bypassing established gatekeepers and cultivating their own ecosystems of influence and revenue. Sherwood, Nicholson, and Marjoribanks (20) note that sports organisations have historically sought to control both the message and the medium, limiting media access, curating public narratives, and reinforcing organisational brand priorities. With the rise of social media, this command-and-control model has eroded. Athletes are no longer dependent on institutional platforms to tell their stories and make them visible to fans and sponsors. During major events, these shifts are particularly visible. Schibblock et al. (21) examined professional alpine skiers’ social media use during the Winter Olympics and World Cup, showing how athletes use personal platforms to craft narratives independent of team or federation messaging. In doing so, they expand their brand reach and influence the way the sport is consumed and understood. Disintermediation also facilitates forms of athlete activism and self-representation that were previously constrained. Ho and Tanaka (22) show how multiracial Japanese athletes Naomi Osaka and Rui Hachimura employ “silent activism” through visual cues and carefully managed self-presentation. These expressions of identity and advocacy are amplified by social media and include themes such as mental health, racial justice, faith, poverty, gender equality, and LGBTQ + inclusion. Bae, Hahn, and Cho (23) analyse the Instagram presence of top-ranked Korean women golfers, revealing a strategic hybridisation of content that balances cultural modesty with cosmopolitan appeal. Athletes in their study negotiate multiple stakeholder interests, illustrating a form of controlled independence where autonomy is asserted without entirely severing ties to traditional structures. Tickell, Sobral, and Meier (24) offer a contrasting view from smaller leagues. Their study of European football's secondary leagues illustrates how organisations lacking resources to compete in the digital attention economy often pivot toward niche content strategies or community-based branding. Across these contexts, disintermediation is associated with athletes’ ability to communicate directly with global audiences, curate personal narratives, and establish revenue streams that operate alongside or outside traditional institutional frameworks.

### Digital platforms and athlete empowerment

The literature identifies digital platforms as transformative forces in the contemporary sport economy, redefining how athletes communicate and monetise their brands, and how they exercise agency across social, cultural, and political dimensions. These platforms have facilitated a redistribution of communicative and commercial power, enabling athletes to speak directly to global audiences. Westerbeek (25) notes that the platform economy has created new dependencies on algorithms, engagement metrics, and proprietary ecosystems that influence the visibility of athletes and how it impacts their relevance and commercial viability. Anderski et al. (26) frame digital platforms as spaces for co-created meaning, where athletes and fans engage in continuous negotiation of identity. Part of that negotiation includes narratives that express values and brand purpose. This process extends beyond text and image to include live interactions, comment threads, and personalised experiences that strengthen emotional connection and brand loyalty. Bleier, Fossen, and Shapira (27) place these dynamics within the broader creator economy, where distinctions between athletes and influencers blur. They highlight monetisation structures such as branded content and platform-native revenue tools, showing how different platforms encourage distinct branding behaviours and community dynamics. This includes Instagram for visual curation, TikTok for virality, YouTube for depth, and Twitch for immediacy. Reconfigurations of athlete market value are a tangible outcome of platformisation. Brison and Geurin (28) show that social media engagement metrics have become central in determining endorsement potential, often equalling the weight of on-field performance. Athletes with high interaction rates, regular posting habits, and perceived authenticity are rated more favourably by sponsors. Lim et al. (29) identify a measurable link between NFL players’ social media activity and on-field performance metrics, suggesting that digital presence may influence both brand equity and public perception. Su et al. (30) document the rise of parasocial relationships, where fans feel emotionally connected to athletes they do not personally know. These connections have gained particular value under Name, Image, and Likeness (NIL) legislation in the USA (54), which allows college athletes to monetise their social capital. Psaltidis (31) characterises the modern athlete as a “post-athlete media performer”, whose identity exists within a continuous cycle of visibility that makes them more relatable. Authenticity emerges as a recurring theme. Nichols and Shapiro (32) find that audiences favour athletes who demonstrate “relatable realness”, especially in endorsement contexts. Wiebach (33) identifies a “digital double bind” for female athletes, who must reconcile gendered expectations with performance visibility. Yoo (34) shows how South Korean athletes use visual self-presentation to manage national identity and combine this with celebrity status and fan expectations. Risks are also documented. Geissler et al. (35) forecast a sport media landscape shaped by algorithmic optimisation that may lead to attention scarcity and platform monopolisation. This raises concerns about dependence on third-party systems. Qi et al. (36) highlight the mental health implications of digital labour and constant visibility, including boundary erosion and surveillance dynamics. The literature notes that monetisation models include branded content, affiliate links, crowdfunding, and direct-to-fan subscriptions, with earnings tied to engagement metrics. The increasing integration of generative AI introduces additional complexities in authorship and content control.

### Generative AI in branding and content creation

The literature identifies generative artificial intelligence (AI) as a technological shift that redefines the boundaries of athlete identity and the nature of branding labour. Generative AI tools such as automated video editing, synthetic voiceovers, personalised AI-driven messages, and deepfake-enabled fan interactions, have entered the athlete's branding toolkit. These developments extend the capabilities of digital platforms, enabling new forms of fan intimacy while raising ethical and psychological questions. Gao et al. (37) demonstrate that AI can streamline content production, personalise messages at scale, and optimise digital advertising in real time. Athletes can automatically generate highlight reels and offer this to segmented audiences by preference, and tailor messages to sub-groups, thereby increasing engagement without proportionally increasing workload. Li and Huang (38) map AI integration across the sport ecosystem, including augmented fan experiences and interactive merchandise. AI is also used for engagement analytics, fan profiling, sentiment monitoring, and performance optimisation, prompting governance concerns about data sovereignty and control over digital footprints. Li and Huang (38) further note that AI is increasingly embedded within platform infrastructures, making it invisible yet ever-present in shaping how athlete brands are experienced. AI enables hyper-targeted marketing and low-cost content generation, lowering the barriers for athletes without access to elite media resources. This democratising effect allows emerging athletes to compete with superstars in the attention economy by delegating parts of their brand work to AI systems. The literature also documents potential risks to authenticity and trust. Mohammadi et al. (39) find that trustworthiness is essential to athlete–fan–brand relationships, and that credibility can be undermined when audiences suspect automation or inauthentic voices in communication. Breves et al. (40) report that perceived fit between athlete and brand declines when content appears overly polished or impersonal. Xu and Baghaei (41) examine the use of AI-generated avatars and deepfakes in sport, noting potential for enhanced entertainment but also ethical concerns about misrepresentation and emotional manipulation. Carrio Sampedro (42) warns of algorithmic deception, where fans engage with synthetic content without being aware it is not human generated. Ownership of AI-generated content emerges as another concern. Carlsson-Wall and Newland (43) and Berkani et al. (44) identify blockchain as a potential mechanism for verifying content origin, authenticating intellectual property, and enforcing royalties for AI-generated merchandise or NFTs. However, legal frameworks are underdeveloped, and many athletes, especially without institutional support, lack the capacity to navigate these systems. Kapoor et al. (45) observe that social media research often overlooks questions of algorithmic ownership and platform bias. From a strategic perspective, Momenifar et al. (46) categorise AI sport marketing applications into data analytics, sentiment detection, creative automation, and virtual branding. They argue that competitive advantage lies in integrating AI with human-led storytelling. They also highlight a generational divide, with younger, digitally native athletes more adept at using AI for brand extension while maintaining perceived control, and older athletes or legacy organisations slower to adapt.

### Emerging athlete-led business models

The literature identifies a convergence of digital autonomy and generative technology where platform-based business structures are the drivers of new athlete-led models in sport. Athletes are increasingly taking on roles as founders, investors, rights-holders, and platform operators, creating ventures that challenge the historically centralised nature of sport commercialisation. McLeod (47) examines the viability of athlete-owned leagues, using basketball and football case studies to show how these competitions reclaim governance and revenue control, and also media rights from traditional sport structures. Although such leagues face operational and financial barriers, they represent an athlete-driven approach to building equity and ownership rather than simply generating income. Blockchain technologies further accelerate decentralisation. Taherdoost and Madanchian (48) describe how blockchain enables secure, transparent ownership of digital assets, facilitating direct athlete–fan engagement (55). Smart contracts, tokenised sponsorships, and non-fungible tokens (NFTs) allow athletes to monetise moments, merchandise, and even fractional rights to future revenue. Wilson, Karg, and Ghaderi (49) outline the risks and opportunities of NFT-driven sport economies, noting that while market volatility is a concern, the technology allows athletes to bypass centralised platforms and create independent economic ecosystems, such as fan access tokens, digital memorabilia, and exclusive content drops. Yadav et al. (50) link blockchain's decentralisation to social media dynamics, showing how distributed ledger systems can safeguard athlete data sovereignty and enable micro-investments by fans which might play a role in reshaping trust between athlete and audience. The transparency of distributed ledgers reduces reliance on centralised authorities such as clubs, agencies, or platforms, in order to validate transactions. This allows athletes to assert ownership over digital identities and commercial assets while enabling fans to participate in verified, token-based exchanges. Stegmann, Nagel, and Ströbel (4) note that value co-creation plays an increasing role in sport marketing, with fans actively shaping athlete brands and narratives into commercial strategies. Sturm (51) frames this participation as a double-edged sword: while it can foster authentic, grassroots brand ecosystems, it also creates risks of surveillance, performative activism, and blurred boundaries between private and public spheres. Ströbel and Germelmann (52) emphasise that managing an athlete-led brand in this environment requires expertise in digital infrastructure, branding strategy, intellectual property law, and stakeholder governance. Athletes must act as media entrepreneurs, understand rules and legislation around data and be aware of and sentient to cultural and social dynamics in roles that demand skills beyond athletic performance or sponsorship negotiation. Without supportive infrastructure such as digital rights training, incubator programs, professional support staff and transparent content marketplaces, access to these models may remain limited to elite athletes.

## Discussion

### Athlete branding and self-production

Viewed through value co-creation theory (2, 3), the athlete branding practices identified in the literature represent more than a one-way projection of image; they are co-produced with multiple stakeholders. Fans engage in interpreting behind-the-scenes content, sharing philanthropic initiatives, and amplifying lifestyle narratives, thereby actively shaping the brand's meaning. This co-creative process is evident in the strategic use of authenticity which is a quality that emerges not only from the athlete's own storytelling but from the reciprocal interactions and feedback loops facilitated by digital platforms (5, 6). From a platform capitalism perspective (1), while these digital environments empower athletes to bypass traditional gatekeepers, they also embed the brand within corporate-controlled infrastructures. The very algorithms that allow event-based “superboosting” can also determine and limit which audiences see an athlete's content. This introduces structural dependencies. Athlete reach and monetisation potential are subjected to the opaque, profit-driven logic of platform owners. The evidence on gendered branding strategies (16, 17) further suggests that the co-creation process is not uniformly empowering. Gender norms and audience expectations filter the kinds of content that are rewarded or penalised by both fans and algorithms. This aligns with platform capitalism's observation that platform design can reinforce existing social hierarchies, even while offering new forms of visibility. The literature on emotional labour and self-brand connection (18) points to another implication, that co-creating a brand in this way demands sustained personal investment. Athletes must continually manage a balance between personal authenticity and market-oriented storytelling, with fan intimacy functioning as both a differentiator and a workload. Finally, the transnational fan relationships documented by Jara Pazmino and Pack (19) show that co-creation extends across geographic and cultural boundaries, particularly when narratives are framed around vulnerability or shared social experiences. For sport managers, this highlights an opportunity but also a challenge in facilitating brand strategies that honour authenticity while recognising the commercial and governance implications of platform-mediated athlete–fan relationships.

### Disintermediation in sport business

Viewed through the lens of platform capitalism (1), disintermediation is not the disappearance of intermediaries, but rather a reconfiguration of gatekeeping power. Traditional organisational structures are supplanted by platform owners whose algorithms determine what is visible, what is worth attention or money, and which audiences are most likely to be reached. Athletes may bypass federations or clubs, but they remain embedded within corporate-controlled infrastructures that shape their brand trajectories in opaque ways. This reflects a shift in dependency rather than pure liberation. From a value co-creation perspective (2, 3), disintermediation offers unprecedented opportunities for athletes to co-produce brand meaning and cultural value directly with fans and sponsors. Studies such as Bae et al. (23) show how curated self-presentation can be a site of negotiated meaning-making, balancing global market appeal with local identity markers. Ho and Tanaka's (22) findings illustrate how activism becomes part of this co-creation process, as audiences interpret and amplify athletes’ visual cues and narrative choices. However, the process also commercialises athlete personhood and what Ho and Tanaka describe as the paradox of authentic commodification, where the more an athlete owns their voice and values, the more marketable they become. In platform capitalism terms, this authenticity is both a differentiator and a monetisable asset, raising questions about how far self-expression can be governed or protected within existing institutional frameworks. For global stars, disintermediation can mean strategic independence, diversified revenue, and enhanced social influence. For smaller leagues and less resourced organisations, as Tickell et al. (24) suggest, it can mean marginalisation. Without integration into athlete-led digital ecosystems, these institutions risk obsolescence in the eyes of digitally native fans. This creates a governance dilemma: should sport organisations position themselves as co-creators in athletes’ branding ecosystems or attempt to reclaim control at the risk of alienating both athletes and audiences? In practical terms, disintermediation demands new sponsorship activation models, updated media access policies, and athlete–organisation partnership frameworks that acknowledge the co-production of value. Governance models will need to address not only institutional integrity but also the realities of platform dependence and the co-ownership of brand narratives.

### Digital platforms and athlete empowerment

Through the lens of value co-creation theory (2, 3), digital platforms act as arenas where athletes and stakeholders collaboratively construct brand meaning. Anderski et al.'s (26) depiction of athlete–fan identity negotiation illustrates how audiences are not passive recipients but active participants in shaping an athlete's symbolic capital. Parasocial relationships (30) and live interactions become mechanisms for sustained co-creation, generating both emotional and commercial value. The NIL context further underscores that co-created brand value can be monetised directly by athletes without institutional mediation. From a platform capitalism perspective (1), the same platforms that enable direct-to-fan empowerment also impose structural dependencies. Algorithmic ranking systems and monetisation rules determine visibility and earning potential. As Westerbeek (25) and Geissler et al. (35) warn, these dependencies create conditions in which autonomy is conditional: athletes remain beholden to platform logics that are opaque, proprietary, and profit driven. The “digital double bind” identified by Wiebach (33) can be read as a form of algorithmically mediated labour, where gender norms intersect with platform expectations, amplifying pressures to perform both authenticity and marketability. The recalibration of athlete market value documented by Brison and Geurin (28) and Lim et al. (29) reflects a structural shift in how sponsorship decisions are made. Engagement metrics have become currency, sometimes superseding sporting achievement. In platform capitalism terms, this means that market value is increasingly defined by data-driven indicators of attention rather than traditional performance hierarchies. This shift can enhance commercial opportunities for digitally skilled athletes but also risks penalising those less active online, creating new inequalities in visibility and sponsorship access. The emergence of the “post-athlete media performer” (31) epitomises the entanglement of performance identity with the attention economy. Visibility becomes an imperative, and content creation a continuous labour process that extends beyond competition schedules. In value co-creation terms, the athlete's brand is co-maintained in real time through interactions with fans and partners; in platform capitalism terms, it is continually measured, optimised, and commodified. The growing role of generative AI intensifies these dynamics, enabling new modes of content production but also introducing fresh risks around authorship and authenticity. For governance and policy, this duality of empowerment and dependency raises critical questions: how can sport organisations support athletes’ digital agency while safeguarding their wellbeing and negotiating with platform owners? Practically, the findings suggest that athlete digital strategies should incorporate diversification across platforms, conscious audience co-creation, and protective boundaries to mitigate algorithmic dependency and mental health risks.

### Generative AI in branding and content creation

From a value co-creation perspective (2, 3), generative AI can be seen as a new partner in the co-production of athlete brand meaning. Gao et al. (37) and Li and Huang (38) illustrate how AI enables scaled personalisation, allowing athletes to engage multiple fan segments simultaneously and to tailor narratives that resonate differently across communities. This automation of brand intimacy expands the co-creative capacity of the athlete–fan relationship but also alters its dynamics: the “human touch” in co-creation is now mediated and sometimes replaced by code. From a platform capitalism viewpoint (1), generative AI deepens athlete dependence on platform-owned technologies and datasets. When AI tools are embedded within proprietary ecosystems, as Li and Huang (38) describe, athletes’ ability to produce and distribute content is contingent on the operational rules and algorithmic priorities of the platform owners. This shifts a degree of creative authorship and brand control from the athlete to the platform, even when the surface narrative appears athlete led. The tension between technological efficiency and emotional resonance, noted by Mohammadi et al. (39) and Breves et al. (40), reflects a core dilemma that athletes are expected to deliver authentic engagement while operating within environments that reward optimisation and automation. In co-creation terms, the fan's perception of “realness” is a co-produced outcome, easily disrupted when synthetic cues undermine the relational bond. The risks multiply with synthetic media (41, 42), where deepfakes and AI-generated avatars challenge the ethical foundations of athlete–fan trust. Blockchain authentication (43, 44) offers potential safeguards, but in platform capitalism terms, these solutions may still operate within centralised platform infrastructures, limiting true decentralisation. Moreover, as Kapoor et al. (45) caution, questions of content ownership and algorithmic bias remain underexplored in sport contexts. Strategically, Momenifar et al.'s (46) emphasis on integrating AI with human-led storytelling aligns closely with the concept of what Westerbeek (53), manuscript under review) has called “augmented authenticity”, a hybrid branding strategy where AI enhances but does not replace human expression. This approach offers a pathway for athletes to maintain narrative coherence, emotional truth, and ethical clarity while leveraging the scalability and creative potential of generative technologies. Those able to strike this balance may lead in shaping the next frontier of sport branding, co-creating value with fans while navigating the power asymmetries and dependencies inherent in the platform economy.

### Emerging athlete-led business models

From a value co-creation perspective (2, 3), athlete-led business models represent a shift from transactional consumption of sport to participatory production. Fans become stakeholders who not only consume but also help shape athlete ventures through direct investment through tokenised engagement and collaborative brand storytelling. This participatory logic is evident in blockchain-enabled micro-investments (50) and the narrative co-production described by Stegmann et al. (4). These models redefine the athlete–fan relationship from one of passive admiration to active economic and cultural partnership. Through the lens of platform capitalism (1), these innovations also reveal underlying dependencies. While blockchain and NFTs appear to decentralise economic power, they often operate on centralised exchanges or platform infrastructures that impose their own governance rules and monetisation structures. Athlete-owned leagues (47) can bypass federation control, but still require integration with media rights platforms, streaming services, and digital payment providers. All of these are themselves centralised entities in the platform economy. The risks of fan hypervisibility and performative activism (51) illustrate how the same mechanisms that enable value co-creation can also become instruments of brand pressure. In platform capitalism terms, algorithmic incentives can encourage athletes to align with certain causes or content types for visibility, regardless of personal conviction. This risks turning empowerment into a form of self-exploitation, where authenticity is instrumentalised for market gain. The hybrid nature of these business models where elements of a content studio, personal brand, community hub, and venture capital entity are combined, reflects the evolving role of the athlete as a multi-sector entrepreneur. In value co-creation terms, this diversification expands the scope of collaborative touchpoints with fans and sponsors. In platform capitalism terms, it multiplies the points of dependency on digital infrastructures and algorithmic mediation. Ultimately, these athlete-led models signal a recalibration of power within the sport economy: away from centralised governing bodies and towards athlete-controlled ecosystems. Yet their success depends on building governance frameworks that protect athlete autonomy, provide equitable access to digital tools, and negotiate the balance between entrepreneurial opportunity and platform dependency. The challenge for policy and practice will be ensuring that these models do not simply replace one set of gatekeepers with another but genuinely redistribute value and agency across the sport ecosystem.

## Conclusion

This review has mapped a decisive reconfiguration of the sport business landscape: the rise of the autonomous, self-driving, entrepreneurial, and digitally fluent athlete. Across five interlinked domains: athlete branding and self-production, disintermediation in sport business, digital platform empowerment, generative AI in content creation, and emerging athlete-led business models, the literature converges on a common finding, that the centre of gravity in sport governance, communication, and commercialisation is shifting from institutional actors to athletes themselves. Enabled by digital platforms that are amplified by generative AI, and driven by natural high-performance attitude, athletes are now able to curate their own narratives and develop alternative revenue models that challenge the traditional monopoly of federations, leagues, and clubs. Disintermediation processes have given athletes direct access to sponsors and audiences and platform technology has allowed them to transform these relationships into sustained communities of cultural and economic value. Generative AI extends this capacity, acting as both a creative accelerator and a strategic risk, while blockchain and decentralised commerce introduce the possibility of athlete-owned ecosystems. From a value co-creation perspective, these shifts redefine the athlete-fan relationship as a participatory process in which brand meaning, advocacy, and commercial value are produced collaboratively. From a platform capitalism perspective, they also reveal structural dependencies. While athletes may bypass traditional gatekeepers, they remain subject to algorithmic visibility, and they depend on the platform monetisation rules and data ownership arrangements they do not control.

The implications are structural and urgent. Governing bodies must evolve from control-oriented institutions to facilitators of athlete-led innovation, co-developing governance frameworks that protect both institutional integrity and athlete autonomy. Sponsors must move from transactional endorsements to genuine partnerships that align with athlete-led narratives and data rights. Athletes and their representatives will need to develop strategies that combine platform diversification with the need to integrate technology that also assists in ethical decision making. Beyond ethical technology consideration is the need to proactively manage intellectual property. Without such adjustments, sport organisations risk accelerating their own legitimacy crisis, as athletes gain the means to construct independent commercial futures or disengage from institutional pathways altogether. The transformation described in this review is systemic. Athletes are no longer peripheral to the sport economy. Athletes are becoming primary architects and value creators, and they mediate cultural and social causes and values. The next decade will determine whether sport governance adapts to this reality or becomes peripheral to the new centres of influence in global sport.

### Future research directions

This review underscores that the athlete brand has moved far beyond a subset of sport marketing to become a disruptive force that is reshaping the governance dynamics of sport. Yet significant questions remain under-theorised and under-examined. There is a pressing need to investigate governance models that embed athlete agency both structurally and procedurally. Comparative studies could examine the effectiveness of arrangements such as player unions, athlete advisory boards, or co-ownership structures across different sports and regions, providing a clearer understanding of how governance can adapt to athlete-led innovation. At the same time, the rapid integration of generative AI into athlete branding calls for sustained inquiry into its ethical and legal implications, particularly around identity, consent, content ownership, data extraction, fan segmentation, and algorithmic visibility. The role of synthetic media in shaping brand credibility warrants critical scrutiny, especially as parasocial relationships between athletes and fans are increasingly mediated by automation. The various impacts of digital labour (mental, emotional, commercial) also require deeper exploration, especially where online hate speech, constant visibility, and the conflation of performance and popularity metrics create new forms of pressure. Platform design and algorithmic curation play a pivotal role in determining which athletes gain or lose visibility, raising questions about inequality between those with institutional backing and those in under-resourced or marginalised contexts. Relatedly, blockchain-enabled engagement models that include tokenised micro-investments, NFT-based community building, and distributed data ownership require further exploration in regard to their scalability and sustainability. It also remains to be seen how inclusive these models can and will be. Future work should also turn attention to fan communities not only as cultural audiences but as coordinated commercial and political actors capable of influencing governance decisions and market outcomes. Understanding these communities as stakeholders reframes the athlete–fan relationship as one of shared agency and reciprocal influence. Finally, there is a need for cross-disciplinary inquiry into the tension between the opportunities for value co-creation offered by digital and decentralised tools and the structural dependencies generated by platform capitalism. The challenge for research, policy, and practice will be to identify strategies that protect athletes from exploitation while enabling them to maximise the shared value that these evolving ecosystems make possible. Sport in the digital era will not be defined by the absence of institutions, but by the redistribution of authority and influence, making it essential to design governance systems that reflect this complexity.
